# Screening of Depressive Symptoms in a Russian General Population Sample: A Web-based Cross-sectional Study

**DOI:** 10.2174/1745017902117010205

**Published:** 2021-12-22

**Authors:** Andrey Alexandrovich Kibitov, Alexander Sergeevich Rakitko, Evgeniy Dmitirevich Kasyanov, Grigoriy Viktorovich Rukavishnikov, Kira Alexandrovna Kozlova, Valeriy Vladimirovich Ilinsky, Nikolay Grigor’evich Neznanov, Galina Elevna Mazo, Alexander Olegovich Kibitov

**Affiliations:** 1 V.M. Bekhterev National Medical Research Center for Psychiatry and Neurology, Bekhtereva str., 3, Saint-Petersburg, Russia; 2 Genotek Ltd., Nastavnicheskiy Lane, 17-1, Moscow, Russia; 3 Lomonosov Moscow State University, GSP-1, Leninskie Gory, Moscow, Russia; 4 First Pavlov State Medical University of St. Petersburg, L'va Tolstogo str. 6-8. Saint Petersburg, Russia; 5 Serbsky National Medical Research Center on Psychiatry and Addictions, Kropotkinsky lane, 23, Moscow, 119034, Russia

**Keywords:** Mental health, Depression, Screening, Survey, Internet, Population health

## Abstract

**Background and Objective:**

Web-based screening of depressive symptoms in general non-clinical population can provide better insights into actual prevalence of depressive symptoms and associated risk factors. To study the current prevalence of depressive symptoms in Russian non-clinical population we conducted screening using an online survey based on Depression subscale of Hospital Anxiety and Depression Scale (HADS-D).

**Methods:**

The online survey covered 2610 Russian-speaking respondents and included HADS-D, questions about sex, age and presence of cardiovascular diseases (CVD) diagnoses or symptoms in respondents.

**Results:**

The proportion of respondents with depressive symptoms, estimated by online HADS-D, was 14.4% (11.5% - at subclinical level, 2.9% - at clinical level). The overall HADS-D score was higher in women (p=0.003), in young individuals under 30 y.o *vs*. participants over 42 y.o. (p=0.004) and in individuals with self-reported CVD symptoms (p=0.00002). Linear regression analysis showed that self-reported CVD symptoms increase HADS-D score (p<0.001), but male sex (p=0.002) and older age (p<0.001) decrease it. Logistic regression showed that CVD symptoms increase the risk of depressive symptoms by HADS-D (p=0.033, OR=1.29), but older age (p=0.015, OR=0.87) and male sex (as a trend, p=0.052, OR=0.80) decrease this risk.

**Conclusion:**

Online survey based on HADS-D showed new patterns of depressive symptoms prevalence in Russian non-clinical population. Depressive symptoms prevalence did not differ between men and women and was higher among young people. The reported association between depressive symptoms and CVD was confirmed.

## 1 INTRODUCTION

Depression is one of the most common mental disorders. According to the World Health Organization, it is considered one of the leading causes of disability and contributes significantly to the global burden of the disease [1]. A large number of studies on depression epidemiology are based mostly on the prevalence of the depression diagnosis, using clinical diagnostic criteria [2]. Less attention was paid to the prevalence of depressive symptoms at a subclinical level in the general non-clinical population (that is, among people who did not seek medical help for depression). However, this data can provide more insights into actual prevalence and risk factors associated with this condition.

Validated self-administered screening scales are effective tools for collecting data on the presence of depressive symptoms. It should be noted that screening tools are designed only to detect symptoms of depression, but not to ascertain a clinical diagnosis. Moreover, data on the prevalence of depressive symptoms acquired with the use of screening tools have been shown to be higher than the prevalence of depression as a clinical diagnosis [3]. Nevertheless, its use can simplify data collection and increase the sample size without losing the quality of depressive symptoms detection. One of the candidate tools for this purpose is the Hospital Anxiety and Depression Scale (HADS) [4]. The data accumulated over the years of using HADS have shown that this scale is a fairly reliable tool for screening anxiety and depression in general practice patients [5]. HADS includes two subscales - anxiety (HADS-A) and depression (HADS-D). This allows depressive symptoms to be assessed separately using only HADS-D. In addition, due to its simple structure HADS is suitable for online use, which will simplify the data collection procedure, potentially preserving the accuracy of the tool in its ability to detect depressive disorders in the general population. To our knowledge, an online survey based on HADS-D has never been used before for depressive symptoms screening in the general population.

There is evidence in the literature that the use of online surveys has a number of advantages over traditional “paper-based” data collection, which involves direct contact between researchers and participants. Web-based data collection has been shown to be a more cost-effective solution than “traditional” methods. It can reduce the cost of participants recruiting without losing the quality of collected data [6]. Moreover, the critical role of collecting and analyzing big data in medicine makes online questionnaires the main method of phenotyping large cohorts in various national registries, biobanks and genetic databases [7].

It has been suggested that uneven Internet access even in highly industrialized countries may lead to selection bias and, therefore, impair the representativeness of the sample in epidemiological studies. However, this bias does not always lead to a significant change in the results of epidemiological studies [8]. It has also been shown that web-based data collection does not impair the accuracy of the assessment of depressive symptoms in the general population [9, 10]. Moreover, the absence of direct contact between participants and researchers during online surveys may allow the inclusion of people who would not participate in any other study with “direct” data collection. Research on drug addictions has shown that web-based data collection is an effective method for including “hidden” populations into samples [11, 12]. Thus, the use of online surveys can be the most appropriate method for assessing the prevalence of depressive symptoms in the general nonclinical population.

The last study on the prevalence of depressive symptoms in Russia was carried out in 2012-2013 using HADS within the framework of the ESSE-RF epidemiological study [13]. It should be noted that in this study, HADS was used in a “paper” format and was a part of extensive medical examination with a variety of laboratory and instrumental methods. Recent epidemiological data indicate an increase in depression prevalence over time [14]. Accordingly, the current prevalence of depressive symptoms in the Russian population may differ significantly from 2012-2013.

Based on the data published to date, it is also possible to highlight some patterns of depressive symptoms prevalence in various population groups. In particular, it is known that the prevalence and severity of depressive disorders are higher in women compared with men [15]. Depression prevalence is also heterogeneous in different age groups [16]; a higher prevalence of depression has also been demonstrated among people with cardiovascular diseases (CVD) [17]. These patterns can also change over time, as has been shown in other populations [18, 19]. Thus, for a better understanding of the current epidemiological situation, it is necessary to study not only the prevalence of depressive symptoms but also its associations with the above-mentioned factors.

Considering the data on changes in depression epidemiology over time, a study on the current prevalence of depressive symptoms in the general Russian population is needed. Thus, we conducted depressive symptoms screening in the Russian non-clinical population using an online survey based on HADS-D to study the current prevalence of depressive symptoms and its association with factors such as sex, age and the history of CVD.

## 2 MATERIALS AND METHODS

This cross-sectional study was carried out as a part of the “GWAS-based and clinically validated system of polygenic risk scores for major depression in the Russian population” project by the Russian National Consortium for Psychiatric Genetics [20]. The clients of Genotek Ltd., provider of genetic testing services in the Russian Federation, participated in this study. Data were collected during 2019-2020 by an online survey on the Internet portal of Genotek Ltd. All clients of Genotek Ltd. over 18 years old were invited to participate in the study. Respondents who agreed to participate and have provided written informed consent were included in the study. Respondents who did not complete the full questionnaire were excluded from the study. Respondents' personal data was hidden during the study.

The questionnaire was in Russian language and consisted of three blocks. The first block contained questions about sex and age. The second block was the HADS-D, which contained 7 questions with 4 options to answer, assessed from 0 to 3 points depending on the severity of the symptom. The third block consisted of questions about whether the respondent had diagnoses of cardiovascular diseases (CVD) or certain symptoms associated with CVD. The respondents were asked to answer “yes” or “no” to the questions about the presence of the following conditions: “high blood pressure (hypertension)”, “cardiac rhythm disturbances”, “chest pain”, “shortness of breath”, “stroke”, “heart attack”, “lower extremity vessel thrombosis”, “pulmonary embolism”, “varicose veins”. The presence of at least one affirmative answer was the reason for classifying the respondent as a person with “possible CVD”.

To test the differences between the respondents depending on age, groups by age were defined based on the analysis of the age distribution in the studied cohort using quartiles (Q1 = 30, Q2 (Me) = 35, Q3 = 42). Accordingly, four age groups were assigned: up to 30 y.o., 31- 35 y.o., 36-42 y.o, 43 y.o. and older.

We analyzed the differences between groups for two characteristics associated with the HADS-D score for each participant: the mean score (quantitative assessment) and the severity level (categorial assessment) defined by the individual score: no depression (0-7 points), subclinical depression (8-10 points) and clinical depression (11 or more points) [4]. It should be noted that in the context of HADS-D result assessment, the term “depression” stands not for a specific clinical diagnosis but merely for the presence and severity of depressive symptoms.

There are items in HADS-D that may possibly overlap with CVD symptoms, although HADS-D is considered to be relatively free from questions about somatic symptoms. These items include questions about retardation (“I feel as if I am slowed down” and “I feel cheerful”). Therefore, for a more thorough analysis of association between CVD and depressive symptoms we excluded these questions and used a refined version of HADS-D without retardation items (HADS-D WRI) in addition to the normal version of the scale.

To analyze statistical data, the R programming language was used [21]. For the analysis of quantitative variables, the nonparametric Wilcoxon-Mann-Whitney and the Kruskal-Wallis tests were used. Fisher's exact test was used for the analysis of nominal variables. For multiple comparisons, Bonferroni and Holm corrections were applied. Multiple linear regression was used to assess the effects of sex, CVD and age on the HADS-D score. The effects of these factors on the risk of depressive symptoms by HADS-D were assessed using binary logistic regression. The absence of depression by HADS-D (0-7 points) and the presence of depressive symptoms at the subclinical and clinical levels (8 or more points) were chosen as binary outcomes. It is worth noting that a meta-analysis of studies using HADS-D proved that 8 points are the optimal threshold value for detecting the presence of depressive symptoms in people without psychiatric diagnoses [5].

## 3 RESULTS

### 3.1 Sample Characteristics

Overall, 2610 people were included. 51.7% of the sample were women (1349). The average age of the respondents was (M (SD)) 36.79 (9.67) years. The presence of CVD was reported by 30.4% (794) of the respondents.

In a study cohort, the mean HADS-D score was 4,4 (2,8) points. Overall, 14,4% of the sample had depressive symptoms: 2,9% (75) had clinical depression according to HADS-D (11 or more points) and 11,5% (299) – subclinical depression (8-10 points). Respondents with clinical depression were significantly younger compared to participants without depressive symptoms (34.5 (10.5) y.o. vs. 37.0 (9.7) y.o., p = 0.038). There were no age differences between participants with clinical and subclinical depression (p=0.342).

### 3.2 Sex Differences

In a study cohort, men were significantly older than women (p <0.0001). The mean HADS-D score for women was significantly higher (p = 0.003) (Table **1**). Among women, the proportion of respondents with clinical depression (in comparison with the proportion of the no-depression group) was higher compared to men (p = 0.046), but after applying the correction for multiple comparisons, this difference ceased to reach the level of significance (p=0.139). Thus, women, despite a higher mean HADS-D score, did not differ from men in the prevalence of clinical and subclinical depression as assessed by HADS-D. No significant differences were found in the prevalence of CVD (p=0.202) between men and women.

### 3.3 Age Differences

**Fig. (1):**
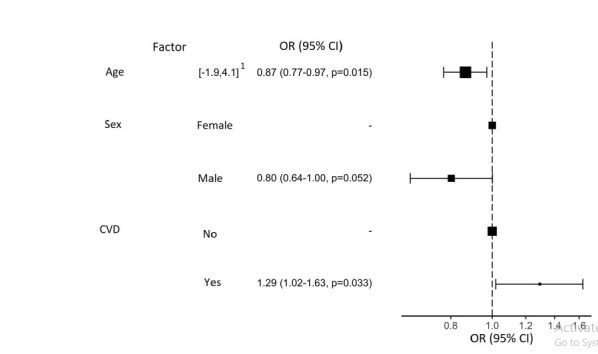
Results of binary logistic regression for assessing the risk of symptoms of clinical or subclinical depression according to HADS-D.

We found that the mean HADS-D score was significantly higher in the group of young respondents (up to 30 y.o.) compared to older respondents (43 and older) (p=0.004) (Table **2** and Fig. **1**), as well as compared with respondents aged 36 to 42, although this difference is only a trend (p=0.082).

Among young respondents (up to 30 y.o.), the proportion of participants with clinically significant depression (when compared with the proportion of participants without depressive symptoms) was significantly higher than in the group of respondents aged 36 to 42 (p=0.00134). Moreover, in the group of older respondents (43 and older), the proportion of respondents with CVD was significantly higher compared to other age groups (p<0.01).

### 3.4 Differences between Respondents with and Without CVD

Among respondents with CVD, the mean HADS-D score (p<0.0001) and the mean HADS-D WRI Score (p=0.002) were higher (Table **3**), but the proportions of participants with subclinical and clinical depression did not differ significantly from those in the group without CVD. Respondents with and without CVD did not differ in the proportion of women (p = 0.202), however, participants with CVD were significantly older (p<0.0001).

### 3.5 Regression Analysis

To assess the possible role of sex, age and the presence of CVD as risk factors for depressive symptoms assessed by HADS-D, regression analysis was performed for the HADS-D score (linear regression) and for the risk of falling into the category of respondents with depressive symptoms, both at clinical and subclinical levels (logistic regression).

Linear regression showed that the presence of CVD significantly increased HADS-D score (p <0.001, β = 0.57, 95% CI [0.33 - 0.80]). On the contrary, male sex (p=0.002, β =-0.33, 95% CI [-0.54 – -0.12]), and older age (p<0.001, β =-0.02, 95% CI [-0.03 – -0.01]) decreased HADS-D score.

Linear regression using HADS-D WRI Score showed that CVD significantly increased HADS-D WRI score (p <0.001, β = 0.34, 95% CI [0.15 - 0.52]). Older age decreased HADS-D WRI score (p=0.001, β =-0.01, 95% CI [-0.02 – -0.01]), but no effects of sex was found (p=0,212).

Logistic regression supports the role of CVD as a risk factor for the symptoms of depression (p=0.033, OR=1.29) and potential protective effects of older age (p = 0.015, OR = 0.87) and male sex (trend, p=0.052, OR=0.80) (Fig. **1**).

1 - spread of values after normalization

## 4 DISCUSSION

Our results showed that the prevalence of depressive symptoms was higher among young participants. Furthermore, there was no difference in the prevalence of depressive symptoms between men and women, although the severity of depressive symptoms was still higher in women. The reported association of depressive symptoms with CVD was confirmed.

According to the results we obtained using the online HADS-D as a screening method for depressive symptoms, the proportion of respondents with depressive symptoms was 14.4% (of which 11.5% were subclinically expressed, and 2.9% clinically expressed). Our estimation is much lower than the results obtained by Shalnova *et al*. in the general Russian population in 2012-2013 during the ESSE-RF study. In this study, the proportion of respondents with subclinical depression (HADS-D ≥ 8) was 25.6%; and with clinical depression (HADS-D≥11) – 8.8% [13]. This inconsistency can be partially explained by the different methodology of the studies (using web-based tools vs. traditional “paper”, different clinical settings) or sample characteristics. But, in spite of that, our results can still highlight the time trend in depressive symptoms prevalence in the Russian population. However, in other studies using HADS, conducted on European populations, the proportion of respondents with 8 or more points was lower – from 11% to 23.5%; the proportion of patients with 11 or more points was comparable, ranging from 5% to 9.6% [22-24]. The mean HADS-D score in our study was 4.4 points, which is also consistent with the data, acquired in the European population, which ranged from 3.0 to 4.7 points [22, 25-28].

In our study, the mean HADS-D score was higher in women. This result is confirmed by numerous studies (also using self-questionnaires) indicating greater severity of depression in women compared to men. In a study by Shalnova *et al*., similar differences in HADS-D scores between sexes have been demonstrated [13]. Sex differences in the severity of depression (assessed using Beck Depression Inventory (BDI) and General Health Questionnaire (GHQ) scales) were also demonstrated in the European population [29]. However, according to the results of our study, the proportion of respondents with depressive symptoms did not differ significantly between men and women. Although data from epidemiological studies based on the formal diagnostic criteria of the DSM or ICD show a higher prevalence of depression in women [30], some HADS studies indicate that there is no such difference. For example, a study by Hinz *et al*., conducted in a German population, demonstrated no significant differences between men and women in terms of both the mean score and the proportion of respondents with depressive disorders [22]. One of the largest HADS studies in the general population, conducted in Norway, also found no significant difference between sexes in the proportion of respondents with depression [27]. In addition, some studies have found even higher rates of depression in men when assessed by HADS-D [24, 31]. Several explanations have been proposed for the observed “reverse” sex pattern of depressive symptoms as measured by HADS-D. Some researchers argue that depression in women is more likely to manifest with somatic symptoms, therefore, the absence of questions about such symptoms in HADS-D may account for decreased levels of depression in women [32]. Other authors demonstrate that this difference may be due to the fact that HADS-D questions are able to identify only one of the possible clinical subtypes of the disease - “anhedonic” depression [27]. At the same time, studies of anhedonia have shown the absence of sex differences. That, in turn, may cause changes in the sex structure of depression when assessed by HADS-D [31]. Furthermore, the majority of the epidemiological data on the higher incidence of depression in women is based primarily on treatment demand. Men are known to seek help less often and, thus, more cases of depression in men remain undiagnosed. It has been shown that treatment demand in men is associated with greater severity of problems than in women [33]. In our study design, respondents did not seek medical care for depression and that could also reduce the observed difference between men and women in depression prevalence. Moreover, this fact may be an advantage of our study, making it possible to identify a more objective picture of the distribution of depressive symptoms in the population outside of their connection with the use of medical care.

We have demonstrated that respondents’ age was also associated with the severity and prevalence of depressive symptoms as assessed by HADS-D. Depression has been considered more common in middle-aged and seniors and that was demonstrated in population studies conducted in the late 20th and early 21st centuries [30]. An increase in the incidence of depressive disorders in older age groups has also been demonstrated in various studies using HADS-D, including the study by Shalnova *et al*. on the Russian population [13, 22, 27]. However, our results indicate greater severity of depressive symptoms in the young age group (up to 30 y.o.), which is consistent with the results of newer studies. For example, in a 2012 study by Patten *et al*., conducted on Canadian adults, there was a 2% decrease in the annual prevalence of depression with each year of life [34]. Similar results were demonstrated in a study by Almas *et al*. conducted on a Swedish population. Authors found that depressed patients (assessed by Major Depression Inventory) were younger than healthy participants [35]. These results can be explained by a significant increase in the incidence of depression in the young age group over the past 15 years. According to the results of the 2017 National Survey on Drug Use and Health study conducted in the United States, the annual prevalence of depression in the 18-25 age group was significantly higher compared to older groups [19].

Our results show that self-reported CVD were also a factor associated with the severity of depressive symptoms when assessed by online HADS-D. This association was shown even using HADS-D without items about retardation, which may possibly overlap with CVD symptoms. Although the proportion of patients with depressive symptoms did not differ significantly among respondents with CVD compared with healthy respondents, logistic regression analysis showed that the presence of CVD increases the risk of developing depressive symptoms (OR=1.29). These results are consistent with the known data on a higher level of depression in patients with CVD in various populations, including the Russian one [13, 30].

To our knowledge, this is the first study on the prevalence of depressive symptoms in the Russian non-clinical population using an online survey based on HADS-D. A large sample of respondents was studied and new data were obtained on the prevalence of depressive symptoms and their association with gender, age and CVD. Considering the effectiveness of an online survey based on HADS-D as a screening method, it can be assumed that this method can also be effective for depression phenotyping in the general population. Currently, there is still a need for developing an effective phenotyping method for genome-wide association studies (GWAS), which will allow the inclusion of large samples in the studies while maintaining the quality of depressive symptoms detection. The use of an online survey based on HADS-D, given its simplicity, cost-effectiveness, and satisfactory clinical validity, can be suitable for studying large population samples with a high level of clinical homogeneity. Thus, this method has the potential for a wider application in genetic (and other biological) research. However, further studies on larger samples are needed to more accurately determine the capabilities and limitations of this tool in depression phenotyping.

This study had several limitations. Although the use of web-based tools can provide valuable results, there is still a risk of sampling bias. It should be noted that our sample was relatively young and older adults could be underrepresented. We did not use diagnostic tools to assess the prevalence of depression as a clinical diagnosis in our sample and were unable to compare these rates with the results of screening with HADS-D. Moreover, HADS-D allows identifying only depressive symptoms that are relevant at the time of testing and does not allow assessing the possible history of depression in the past (lifetime depression). Also, we did not conduct medical examinations of respondents and did not have access to their medical records, and therefore we determined the history of CVD solely on the basis of self-report.

## CONCLUSION

According to the data obtained, the prevalence of depressive symptoms in the Russian non-clinical population has decreased compared to the same data for 2012-2013. At the same time, it was found that depressive symptoms were more associated with young age, and the prevalence of depressive symptoms among men was comparable to that among women (although the severity of depressive symptoms in the latter was still higher). The association between depressive symptoms and CVD was confirmed. Thus, the online survey using HADS-D made it possible to identify new patterns of depressive symptoms prevalence in the Russian non-clinical population.

## Figures and Tables

**Table 1:** Sex differences.

**Sex**	**Men (n=1261)**	**Women (n=1349)**
Age (М(SD)), years	37,6 (9,7)*	36,1 (9,5)
% of respondents with CVD (n)	31,6% (399)	29,3% (395)
**HADS-D**		
*HADS-D* Score (M(SD)), points	4,2 (2,7)	4,6 (2,9)*
0-7 points (no depression), % (n)	87,15% (1099)	84,29% (1137)
8-10 points (subclinical depression), % (n)	10,63% (134)	12,23% (165)
11 and more points (clinical depression), % (n)	2,22% (28)	3,48% (47)

* - p<0,01 between groups

**Table 2:** Age differences.

**Age Groups, Y.o.**	**Up to 30** **(n=682)**	**31-35 (n=698)**	**36-42** **(n=620)**	**43 and Older (n=610)**
% of women (n)	58,7% (400)	51,3% (358)	49,7% (308)	46,4% (283)
% of respondents with CVD (n)	24,5% (167)	28,7% (200)	27,3% (169)	42,3% (258)**
**HADS-D**				
HADS-D Score (M(SD)), points	4,7 (3,0)*	4,5 (2,7)	4,3 (2,7)	4,2 (2,8)
0-7 points (no depression), % (n)	82,84% (565)	85,96% (600)	86,94% (539)	87,21% (532)
8-10 points (subclinical depression), % (n)	12,61% (86)	11,60% (81)	11,45% (71)	10,00% (61)
11 and more points (clinical depression), % (n)	4,55% (31)*	2,44% (17)	1,61% (10)	2,79% (17)

* - p<0,05 *vs.* 43 and older group ** - p<0,01 *vs.* other age groups

**Table 3:** Differences between respondents with and without CVD.

**CVD**	**Without CVD** **(n=1816)**	**With CVD** **(n=794)**
Age (М(SD)), years	35,8 (9,0)	39,0 (10,8)*
% of women (n)	52,5% (954)	49,7% (395)
HADS-D		
HADS-D Score (M(SD)), points	4,3 (2,7)	4,8 (2,8)**
HADS-D WRI Score (M(SD)), points	2,2 (2,1)	2,5 (2,2)**
0-7 points (no depression), % (n)	86,4% (1570)	83,9% (666)
8-10 points (subclinical depression), % (n)	11,1% (201)	12,3% (98)
11 and more points (clinical depression), % (n)	2,5% (45)	3,8% (30)

* - p<0,05 between groups, ** - p<0,01 between groups

## Data Availability

The user agreement (available at https://www.genotek.ru) states that disclosure of individual-level genetic information and/or self-reported Information to third parties for research purposes will not occur without explicit consent. Due to the user agreement the individual level cannot be made directly available to scientific community but have to be accessed indirectly *via* Genotek Ltd.
